# Pulmonary Mucormycosis as the Leading Clinical Type of Mucormycosis in Western China

**DOI:** 10.3389/fcimb.2021.770551

**Published:** 2021-11-22

**Authors:** Junyan Qu, Xijiao Liu, Xiaoju Lv

**Affiliations:** ^1^ Center of Infectious Disease, West China Hospital, Sichuan University, Chengdu, China; ^2^ Radiology Department, West China Hospital, Sichuan University, Chengdu, China

**Keywords:** mucormycosis, pulmonary mucormycosis, diabetes, clinical analysis, clinical outcome

## Abstract

The aim was to better understand the clinical characteristics of patients with mucormycosis in western China. We retrospectively investigated the clinical, laboratory, radiological and treatment profiles of mucormycosis patients during a 10-year period (2010–2019). As a result, 59 proven mucormycosis were enrolled in this study. It was found that 52.5% of patients had worse clinical outcomes. Pulmonary mucormycosis (PM) was the most common clinical manifestation. The most frequent risk factor was diabetes mellitus (38, 64.4%) for mucormycosis patients. Cough (43, 93.5%), fever (24, 52.2%) and hemoptysis/bloody phlegm (21, 45.7%) were the most common manifestations of PM. There were no differences in clinical manifestations, risk factors and laboratory tests between different clinical outcome groups (P>0.05). Lymph node enlargement (30, 65.2%), patchy shadows (28, 60.9%), cavitation (25, 53.3%) and bilateral lobe involvement (39, 84.8%) were the most common on chest CT. Nodule was more common in good outcome group (P <0.05). A total of 48 cases (81.4%) were confirmed by histopathological examination, 22 cases (37.3%) were confirmed by direct microscopy. PM patients were treated with amphotericin B/amphotericin B liposome or posaconazale had better clinical outcomes (P <0.05). In conclusion, PM was the most common clinical type of mucormycosis in China. Diabetes mellitus was the most common risk factor. PM has diverse imaging manifestations and was prone to bilateral lobes involvement. Early diagnosis and effective anti-mucor treatment contribute to successful treatment.

## Introduction

Mucormycosis is a rare, emerging and opportunistic fungal infection with high morbidity and mortality caused by filamentous fungi of the Mucoraceae family, order Mucorales. Mucorales fungi are ubiquitous in nature. Humans are infected mainly by inhaling sporangiospores, occasionally through the ingestion of contaminated food or traumatic inoculation (Prakash and Chakrabarti, 2019; Shariati et al., 2020). The prevalence of mucormycosis varies between developing and developed countries, ranging from 0.01 to 14 per 100 000 population in Europe and India (Bitar et al., 2009; Chakrabarti and Singh, 2014; Ruhnke et al., 2015). With the growth of the number of immunocompromised patients, increased awareness and development of diagnostic techniques, the incidence of mucormycosis is rising (Prakash et al., 2019). According to clinical presentations, mucormycosis is mainly classified as rhino-orbito-cerebral, pulmonary, cutaneous, gastrointestinal and disseminated types (Skiada et al., 2020). The common risk factors of mucormycosis are diabetes, hematological malignancy, use of corticosteroids or immunosuppressants, and trauma (Petrikkos et al., 2014; Jeong et al., 2019). However, the main cause of mucormycosis also varies in different countries. Hematological malignancies are the main cause in countries with high income, while diabetes mellitus (DM) or trauma are the main cause in developing countries (Chakrabarti and Singh, 2014). Diagnosis of mucormycosis is challenging because of the low sensitivity and specificity of clinical diagnostic methods (Skiada et al., 2018).

The mortality of mucormycosis remains high, it may be related to delayed diagnosis, high cost of managing mucormycosis and limited treatment options (Skiada et al., 2018). Previous studies on the characteristics of mucormycosis have been conducted mainly in America, Europe and India (Prakash and Chakrabarti, 2019). Data about mucormycosis from China is sparse. The causative agents of mucormycosis vary with different geographical locations (Prakash and Chakrabarti, 2019), and the epidemiology, the clinical disease pattern of mucormycosis vary from country to country.

To better understand the clinical characteristics of patients with mucormycosis in China, in this retrospective study, we compared the demographic features, clinical presentations, laboratory data, radiographic findings and therapeutic strategies in mucormycosis patients with different clinical outcomes who were admitted to a university hospital from Jan 2010 to Dec 2019 in western China.

## Materials and Methods

### Patients

From Jan 2010 to Dec 2019, the patients with a diagnosis of mucormycosis at hospital discharge were retrospectively reviewed in West China Hospital, Sichuan University, a 4,300-bed academic tertiary hospital in Chengdu, China. According to the European Organization for Research and Treatment of Cancer/Mycoses Study Group (EORTC/MSG) criteria and previous references (De Pauw et al., 2008; Petrikkos et al., 2014; Skiada et al., 2018), inclusion in the final study group required the diagnosis of proven mucormycosis as defined as follows: (1) age ≥ 14 years; (2) clinical manifestations and radiographic findings consistent with mucormycosis; and (3) histological presence of mucormycosis in tissue specimens, and/or broad-based, ribbon-like, non-septate hyphae with right-angle branching filamentous fungi on direct microscopy of clinical specimens, and/or Mucorales species cultured from clinical specimens. Based on clinical presentation and the involvement of the body sites, rhino-orbital-cerebral, pulmonary, cutaneous and disseminated mucormycosis were classified (Jeong et al., 2019). The following data of demographic information, underlying diseases, use of corticosteroid or immunosuppressive agent, clinical manifestations, laboratory data, radiologic findings, diagnostic procedures, therapeutic strategies and clinical outcomes at 90 days were collected.

The study was approved by the Ethics Committee of West China Hospital, Sichuan University. Because all the data in this study were routinely obtained, written informed consent was waived.

### Laboratory Studies

Laboratory tests including complete blood count, blood biochemistry, procalcitionin (PCT), C-reactive protein, T lymphocyte subset, HIV testing, serum (1,3)-beta-D-glucan test (BDG test), and galactomannan test (GM test) were performed. Clinical samples (including blood, sputum, secretions, urine) were aseptically collected and cultured under aerobic or anaerobic conditions. Bacterial species were isolated and identified using MicroScan WalkAway-96 System (Siemens, USA). Fungal culture was performed on Sabouraud dextrose agar (SDA) and incubated at 30°C. All the items were performed in the Department of Laboratory Medicine of our hospital.

### Radiological Assessment

Imaging examinations such as chest computed tomography (CT), abdominal CT, fibroptic bronchoscopy were performed at the discretion of the treating physicians. The CT scans were performed using 64-row multi-slice spiral CT scanner (SOMATOM definition AS+, Siemens) in our hospital. All images were reviewed independently by two experienced radiologists. For patients with PM, chest CT findings including nodule, mass, cavity, patchy consolidation, ground glass opacity, reversed halo sign, lymph node enlargement, pleural effusion and the distribution of the lesion in the lungs were recorded.

### Treatment Strategies and Clinical Outcomes

The patients with mucormycosis were treated with amphotericin B (AmB, 0.5-1 mg/kg per day) (North China Pharmaceutical Co., Ltd., China) or amphotericin B liposome (LAmB, 3-6 mg/kg per day) in accordance with the drug instructions, guidelines (Skiada et al., 2013) and patient tolerance. Posaconazole oral suspension (40mg/ml, 10ml, twice daily) (Patheon Inc, Whitby, ON, Canada) was used in patients who have contraindications to amphotericin B or who cannot tolerate the side effects of amphotericin B like reduced kidney function, electrolyte imbalances, nausea and vomiting. The treatment duration was determined by the treating physicians according to the patient’s treatment response and the size of the focus. Management of patients with diabetes included dietary guidance, tight glucose control with insulin and/or hypoglycemic drugs, multiple flash glucose monitoring daily and treatment of complications. A multidisciplinary consulting team, including diabetes, infectious disease, nutrition, wound therapy, surgery, clinical microbiology, and clinical pharmacy, that offered specialist advice, on-going management. Other underlying diseases of these patients such as immune system disease, chronic lung disease were treated normally and systematically. For bacterial co-infection patients, empirical or targeted antimicrobial therapy were given. Clinical outcomes of patients with mucormycosis were evaluated at 90 days after diagnosis. According to clinical manifestations, laboratory findings and image changes, their clinical outcomes were divided into good and worse outcome. Death or disease progression or persistence were classified as worse outcome. The continuous improvement of clinical symptoms and imaging findings were classified as good outcome.

### Statistical Analysis

Statistical analyses were performed using IBM SPSS Statistics for Windows v.26.0 (IBM Corp., Armonk, NY, USA). The Shapiro-Wilk normality test was used to test the normality of all quantitative variables. Continuous variables with normal distribution were presented as mean± standard deviation (SD) and compared by Student’s t-test. The relationship between categorical variables was assessed using Chi-square test or Fisher’s exact test. A two-tailed P value lower than 0.05 was considered statistically significant.

## Results

### Demographic Characteristics and Clinical Outcomes of Mucormycosis Patients

A total of 68 patients with mucormycosis were admitted to our hospital from January 2010 to December 2019, 59 of whom with proven mucormycosis (mean age 54.75 ± 14.72 years; 44 males) were enrolled in this retrospective study. Pulmonary mucormycosis (PM) was the most commonly observed manifestation (46/59, 78.0%), followed by rhino-orbital-cerebral (9/59, 15.3%) and disseminated mucormycosis (3/59, 5.0%). There was only one case of cutaneous mucormycosis. These patients were classified as pulmonary type and other type mucormycosis based on clinical type. The demographic and clinical outcomes of these patients were summarized in **Table 1**. The ages of patients with PM was higher than that of patients with non-pulmonary mucormycosis (P=0.015). The most frequent underlying disease was diabetes mellitus (38, 64.4%) for mucormycosis patients. More patients with PM had diabetes mellitus than patients with non-pulmonary mucormycosis (P=0.027). A total of 31 mucormycosis patients (52.5%) had worse clinical outcomes, clinical outcome was not obviously different between the two groups (P>0.05).

**Table 1:** Demographic and clinical outcomes in patients with mucormycosis.

Variables		Clinical type		P-value
	All (N = 59)	Pulmonary type (N = 46)	Other type (N = 13)	
**Male**	44 (74.6)	35 (76.1)	9 (69.2)	0.616
**Age (years) (mean ± SD)**	54.75 ± 14.72	57.41 ± 12.40	45.31 ± 18.64	0.015*
**Underlying diseases or risk factors**				
Diabetes mellitus	38 (64.4)	33 (71.7)	5 (38.5)	0.027*
Immune system disease	2 (3.4)	1 (2.2)	1 (7.7)	0.395
Corticosteroid medication or immunosuppressive drugs	4 (6.8)	3 (6.5)	1 (7.7)	1.000
Hematological malignancy	3 (5.1)	1 (2.2)	2 (15.4)	0.119
Solid tumor	2 (3.4)	2 (4.3)	0 (0.0)	1.000
Chronic kidney diseases	3 (5.1)	3 (6.5)	0 (0.0)	1.000
Chronic lung disease	8 (13.6)	7 (15.2)	1 (7.7)	0.671
Chronic liver disease	2 (1.7)	1 (2.2)	1 (7.7)	0.395
Truma	2 (3.4)	1 (2.2)	1 (7.7)	0.395
None	8 (13.6)	6 (13.0)	2 (15.4)	1.000
**Co-infection of other pathogens**				
Bacteria	42 (71.2)	32 (69.6)	10 (76.9)	0.738
Other fungi	16 (23.7)	14 (30.4)	2 (15.4)	0.481
**Clinical outcome**				
Good outcome	28 (47.5)	23 (50.0)	5 (38.5)	0.462
Worse outcome	31 (52.5)	23 (50.0)	8 (61.5)

*P < 0.05.

### Demographic and Clinical Characteristics in Patients With PM

Demographic and clinical features of PM patients with different clinical outcomes were shown in **Table 2**. The most common clinical symptoms of PM patients were cough (43, 93.5%), fever (24, 52.2%) and hemoptysis/bloody phlegm (21, 45.7%). Diabetes mellitus (33, 71.7%) was the most frequent underlying disease. There were no significant difference in the gender, age, clinical symptoms and underlying diseases beween good and worse outcome group (P>0.05). For laboratory results, there was no significant difference between the two groups (P > 0.05).

**Table 2:** Demographic and clinical characteristics in patients with pulmonary mucormycosis.

Variables	All (N = 46) (n,%)	Good outcome (N = 23) (n,%)	Worse outcome (N = 23) (n,%)	P-value
Male	35 (76.1)	5 (21.7)	6 (26.1)	0.288
Age	57.41 ± 12.40	56.00 ± 13.44	58.82 ± 11.38	0.446
**Presenting symptoms and signs**				
Fever	24 (52.2)	13 (56.5)	11 (47.8)	0.768
Cough	43 (93.5)	21 (91.3)	22 (95.7)	1.000
hemoptysis or bloody phlegm	21 (45.7)	9 (39.1)	12 (52.2)	0.554
Chest pain	7 (15.2)	2 (8.7)	5 (21.7)	0.414
Shortness of breath	14 (30.4)	9 (39.1)	5 (21.7)	0.200
**Underlying diseases or risk factors**				
Diabetes mellitus	33 (71.7)	16 (69.6)	17 (73.9)	1.000
Immune system disease	1 (2.2)	0 (0.0)	1 (4.3)	1.000
Corticosteroid medication or immunosuppressive drugs	3 (6.5)	2 (8.7)	1 (4.3)	1.000
Hematological malignancy	1 (2.2)	1 (4.3)	0 (0.0)	1.000
Solid tumor	2 (4.3)	0 (0.0)	2 (8.7)	0.489
Chronic kidney diseases	3 (6.5)	2 (8.7)	1 (4.3)	1.000
Chronic lung disease	7 (15.2)	4 (17.4)	3 (13.0)	1.000
Chronic liver disease	1 (2.2)	1 (4.3)	0 (0.0)	1.000
Truma	1 (2.2)	1 (4.3)	0 (0.0)	1.000
None	6 (13.0)	4 (17.4)	2 (8.7)	0.665
**Laboratory data**				
White blood cell (× 10^9^/L)	9.13 ± 6.84	9.58 ± 9.02	8.69 ± 3.76	0.665
Neutrophil (%)	71.96 ± 12.24	74.42 ± 12.11	69.38 ± 12.10	0.170
Lymphocyte (× 10^9^/L)	1.48 ± 0.67	1.35 ± 0.60	1.62 ± 0.73	0.180
Eosinophil (× 10^9^/L)	0.33 ± 1.17	0.48 ± 1.46	0.19 ± 0.26	0.397
Mononuclear cell (× 10^9^/L)	0.49 ± 0.23	0.47 ± 0.26	0.50 ± 0.20	0.539
Procalcitionin (ng/mL)	0.21 ± 0.49	0.29 ± 0.66	0.12 ± 0.13	0.289
C-reactive protein (mg/L)	70.31 ± 87.94	66.75 ± 97.40	73.17 ± 82.08	0.831
Eerythrocyte sedimentation rate (mm/h)	55.23 ± 24.06	49.00 ± 24.64	60.42 ± 23.31	0.278
CD4+ T lymphocyte (%)	36.94 ± 10.33	37.32 ± 8.00	36.55 ± 12.54	0.771
CD8^+^ T lymphocyte (%)	31.61 ± 9.49	32.92 ± 8.17	30.30 ± 10.80	0.475
CD4/CD8 ratio	1.36 ± 0.77	1.23 ± 0.40	1.48 ± 1.01	0.409
CD4^+^ T lymphocyte (cells/μl)	565.45 ± 288.69	419.83 ± 92.04	740.2 ± 357.47	0.062
CD8^+^ T lymphocyte (cells/μl)	396.82 ± 176.78	338.50 ± 45.12	466.80 ± 253.69	0.250
**Co-infecting agent**				
Bacteria	25 (54.3)	12 (52.2)	13 (56.5)	1.000
Virus	2 (4.3)	1 (4.3)	1 (4.3)	1.000
Fungi	18 (39.1)	10 (43.5)	8 (34.8)	0.763

### Findings of CT Scan in Patients With PM

**Figure 1:**
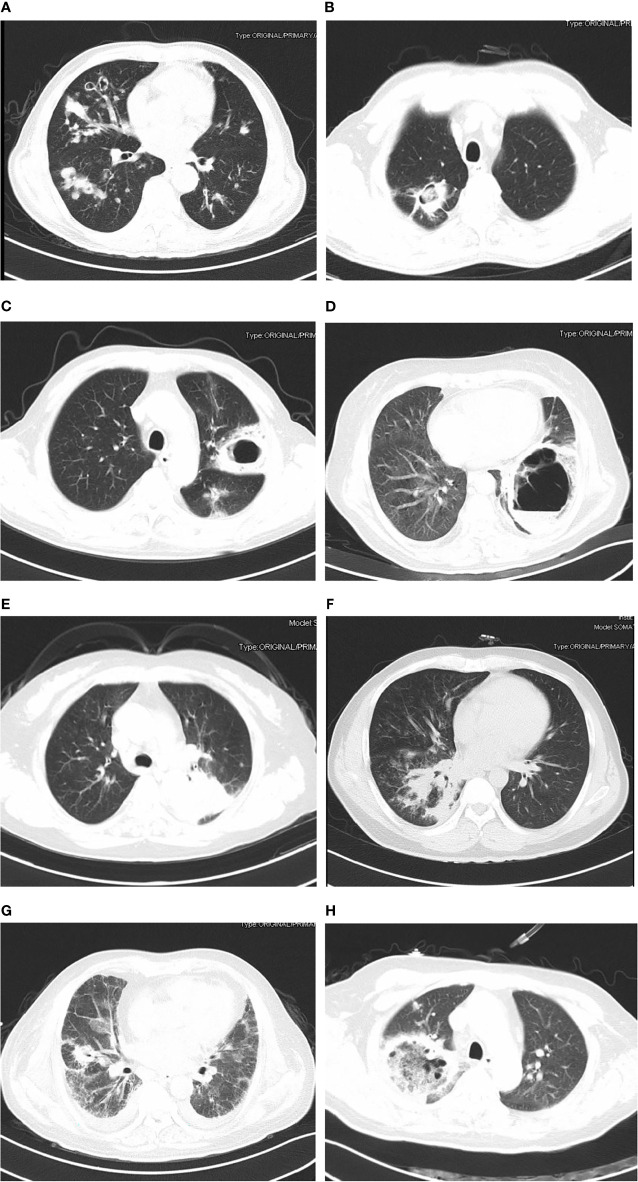
The findings of chest computed tomography of pulmonary mucormycosis (patients with good outcome: **A, B**; patients with worse outcome: **C–H**). **(A)** Multiple pleomorphic lesions in both lungs; **(B)** cavity with muralnodule (*Mucor* co-infecting with *Aspergillus*); **(C)** cavity in left lung; **(D)** thick-walled cavity shadow with gas-fluid level (*Mucor* co-infecting with *Klebsiella pneumoniae*); **(E)** mass shadow in the upper lobe of the left lung; **(F)** patchy shadow, nodules and consolidation in right lung; **(G)** cavity and ground-glass opacity; **(H)** reversed halo sign.

**Figure 2:**
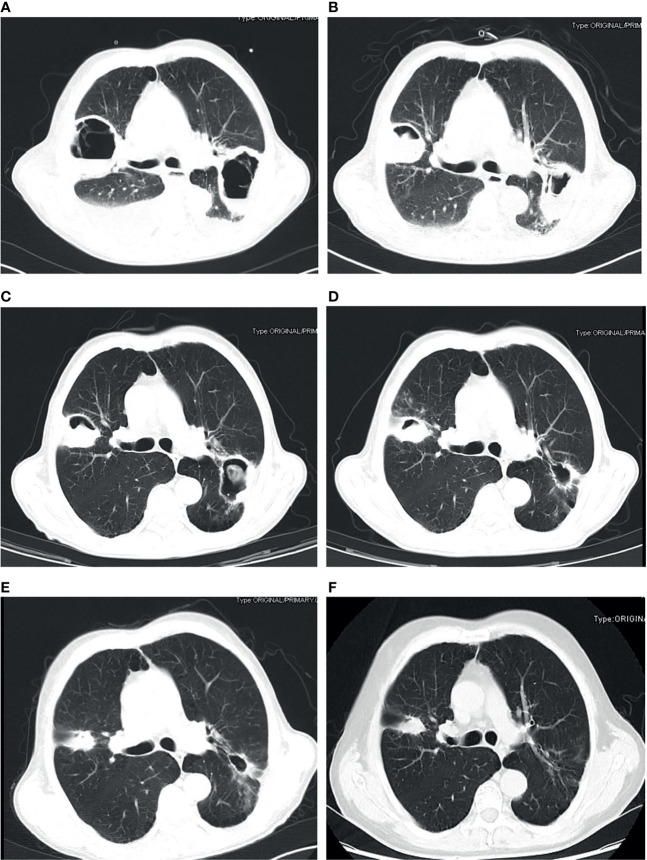
Pulmonary fungal infection (*Mucor* with *Aspergillus*) in a 69-year-old man with uncontrolled diabetes, chronic obstructive pulmonary disease and liver cirrhosis. CT imaging showed thick-walled cavity shadow with gas-fluid level in the right upper lobe and left lower lobe of the lung **(A)**. The lesion was significantly reduced after half a month of treatment with posaconazole **(B)**. The lesion continued to shrink after 40 days **(C)**, three months **(D)**, five months **(E)** and 1 year **(F)** of treatment.

The chest CT features of patients with PM were diverse, as shown in **Figure 1**. The detail of chest CT findings in these patients were shown in **Table 3**. The most common manifestations were lymph node enlargement (30, 65.2%), patchy shadows (28, 60.9%), cavitation (25, 53.3%) and nodules (24, 52.2%). Nodule was more common in good outcome group than in worse outcome group (P <0.05). Lymph node enlargement were more common in patients with worse outcome (P<0.05). Bilateral lungs were involved in 84.8% (39/46) of patients, with the left lower lobe (20, 43.5%) and right lower lobe (18, 39.1%) most commonly involved. There was no significant difference in the lobe of lesion distribution between the two groups (P>0.05). There were 13 patients with *Mucor* and *Aspergillus* co-infection and 1 patient with lung cancer. Based on imaging findings, 12 patients were suspected to be tumors, and 2 patients were suspected to be aspergillosis in the absence of concomitant *Aspergillus*. **Figure 2** showed the changes of chest CT lesions during the follow-up of a patient with pulmonary fungal infection (*Mucor* with *Aspergillus*).

**Table 3:** Chest CT findings of proven patients with pulmonary mucormycosis .

Morphology	All (N = 46) (n,%)	Good outcome (N = 23) (n,%)	Worse outcome (N = 23) (n,%)	P-value
Consolidation	20 (43.5)	9 (39.1)	11 (47.8)	0.767
Nodule	24 (52.2)	16 (69.6)	8 (34.8)	0.038*
Ground-glass opacity	7 (15.2)	5 (21.7)	2 (8.7)	0.414
Mass	15 (32.6)	8 (34.8)	7 (30.4)	1.000
Cavitation	25 (53.3)	10 (43.5)	15 (65.2)	0.139
Patchy shadow	28 (60.9)	14 (60.9)	14 (60.9)	1.000
Fibrosis	17 (37.0)	8 (34.8)	9 (39.1)	1.000
Pleural effusion	22 (47.8)	11 (47.8)	10 (43.5)	1.000
Pleural thickening	17 (37.0)	6 (26.1)	11 (47.8)	0.221
Lymph node enlargement	30 (65.2)	11 (47.8)	19 (82.6)	0.029*
Reversed halo sign	5 (10.9)	2 (8.7)	3 (13.0)	1.000
Lobe of lesion distribution				
Left upper lobe	12 (26.1)	5 (21.7)	7 (30.4)	0.738
Left lower lobe	20 (43.5)	11 (47.8)	9 (39.1)	0.767
Right upper lobe	12 (26.1)	7 (30.4)	5 (21.7)	0.738
Right middle lobe	11 (23.9)	8 (34.8)	3 (13.0)	0.165
Right lower lobe	18 (39.1)	8 (34.8)	10 (43.5)	0.763
Bilateral involvement	39 (84.8)	20 (87.0)	19 (82.6)	1.000

*P < 0.05.

### Diagnostic Procedures and Treatment Strategies

Of the 59 proven patients with mucormycosis, 48 cases (81.4%) were confirmed by histopathological examination, 22 cases (37.3%) were confirmed by direct microscopy. There was no significant difference in the diagnostic procedures between the two groups (P>0.05). A total of 47 patients (79.7%) received treatment. Treatment strategies for these patients with mucormycosis include antifungal drugs, surgery, or both. Most patients (38/59, 64.4%) were treated with amphotericin B/LAmB. More patients with PM were treated with AmB/LAmB (P=0.006) or posaconazale (P=0.004) in good outcome group than in worse outcome group.

For patients with PM, only 3 patients were treated with surgery and LAmB, but there was no statistical difference in clinical outcome (P>0.05). The most common adverse events of AmB/LAmB included renal insufficiency (16/42, 38.1%) and hypokalemia (9/42, 21.4%) in this study. The severity of all adverse events were mild to moderate (grade 1-2) according to the US National Cancer Institute Common Toxicity Criteria (NCI-CTC) (US, National Cancer Institute, 2017). The adverse reaction ratio of AmB/LAmB has not statistical difference between the two groups (P>0.05). As shown in **Table 4**.

**Table 4:** Diagnostic procedures and treatment strategies for proven patients with mucormycosis .

Diagnostic method	All (N = 59) (n,%)	Good outcome (N = 28) (n,%)	Worse outcome (N = 31) (n,%)	P-value
Cluture	13 (22.0)	7 (25.0)	6 (19.4)	0.755
Direct microscopy	22 (37.3)	7 (25.0)	15 (48.4)	0.105
Histology	48 (81.4)	23 (82.1)	25 (80.6)	1.000
Cluture positive only	8 (13.6)	5 (17.9)	3 (9.7)	0.458
Direct microscopy positive only	2 (3.4)	1 (3.6)	1 (3.2)	1.000
Histology positive only	25 (52.4)	14 (50.1)	11 (35.5)	0.260
Cluture+histology	4 (6.8)	2 (7.1)	2 (6.5)	1.000
Direct microscopy+histology	19 (32.2)	6 (21.4)	13 (41.9)	0.105
Cluture+Direct microscopy	1 (1.7)	0 (0.0)	1 (3.2)	1.000
BDG test	3 (5.1)	1/24 (4.2)	2/25 (8.0)	1.000
GM test	3 (5.1)	2/20 (10.0)	1/20 (5.0)	1.000
**Therapeutic strategies**				
**Pulmonary**	All (N=46) (n,%)	Good outcome (N=23) (n,%)	Worse outcome (N=23) (n,%)	P-value
AmB/LAmB	28 (60.9)	19 (82.6)	9 (39.1)	0.006*
Posaconazale	11 (23.9)	10 (43.5)	1 (4.3)	0.004*
LAmB+Surgery	3 (6.5)	1 (4.3)	2 (8.7)	1.000
untreated	11 (23.9)	0 (0.0)	11 (47.8)	0.000*
**Rhino-orbito-cerebral**	All (N=9) (n,%)	Good outcome (N=3) (n,%)	Worse outcome (N=6) (n,%)	P-value
AmB/LAmB	7 (77.8)	2 (66.7)	5 (83.3)	1.000
Posaconazale	1 (11.1)	0 (0.0)	1 (16.7)	1.000
AmB+Surgery	1 (22.2)	1 (33.3)	0 (0.0)	0.333
**Disseminated**	All (N=3) (n,%)	Good outcome (N=1) (n,%)	Worse outcome (N=2) (n,%)	P-value
AmB	2 (66.7)	1 (100.0)	1 (50.0)	NA
untreated	1 (33.3)	0 (0.0)	1 (50.0)	NA
**Cutaneous**	All (N=1) (n,%)	Good outcome (N=1) (n,%)	Worse outcome (N=0) (n,%)	P-value
AmB+Surgery	1 (100.0)	0 (100.0)	0 (0.0)	NA
**Adverse reactions of AmB**/**LAmB**				
**Pulmonary**	All (N=31) (n,%)	Good outcome (N=20) (n,%)	Worse outcome (N=11) (n,%)	P-value
Hypokalemia	7 (22.6)	5 (25.0)	2 (18.2)	1.000
Renal insufficiency	11 (35.5)	8 (40.0)	3 (27.3)	0.698
Phlebophlogosis	1 (3.2)	1 (5.0)	0 (0.0)	1.000
Allergic reaction	1 (3.2)	1 (5.0)	0 (0.0)	1.000
Gastrointestinal toxicity	4 (12.9)	2 (10.0)	2 (18.2)	0.601
Cardiotoxicity	3 (10.7)	2 (10.0)	1 (9.1)	1.000
**Rhino-orbito-cerebral**	All (N=8) (n,%)	Good outcome (N=3) (n,%)	Worse outcome (N=5) (n,%)	P-value
Hypokalemia	1 (1.3)	0 (0.0)	1 (20.0)	NA
Renal insufficiency	4 (50.0)	3 (100.0)	1 (20.0)	NA
**Disseminated**	All (N=2) (n,%)	Good outcome (N=1) (n,%)	Worse outcome (N=1) (n,%)	P-value
Hypokalemia	1 (50.0)	0 (0.0)	1 (100.0)	NA
Renal insufficiency	1 (50.0)	1 (100.0)	0 (0.0)	NA
Gastrointestinal toxicity	1 (50.0)	0 (0.0)	1 (100.0)	NA
Agranulocytosis	1 (50.0)	0 (0.0)	1 (100.0)	NA

AmB, amphotericin B; BDG test, (1,3)-beta-D-glucan test; GM test, galactomannan test; LAmB, liposomal amphotericin B.

*P < 0.05; NA, Not Applicable.

## Discussion

Mucormycosis is rare, neglected, and associated with high mortality rates. The study found that 52.5% of patients with mucormycosis had poor clinical outcomes. Which is similar to previous research results (Jeong et al., 2019). PM was the most common form of mucormycosis, accounting for 78.0% of all the patients with mucormycosis. Although previous studies have reported that rhino-orbital mucormycosis was the most common clinical type (Patel et al., 2020), PM maybe more common in China (Peng et al., 2019). This study also found the most common underlying disease was diabetes mellitus. Previous studies have shown that PM mainly occurred in patients with hematological malignancies, while rhino-orbital mucormycosis mostly occurred in patients with diabetes mellitus (Skiada et al., 2011; Chakrabarti and Singh, 2014). However, our results were consistent with those from China (Peng et al., 2019). Perhaps because the number of diabetes patients in China is much larger than that of hematological malignancies (Liu et al., 2019; Khan et al., 2020), the underlying disease of more mucormycosis patients is diabetes. The high prevalence of diabetes in China and endemic mucor species different from other countries may also be the reasons for the high incidence of PM in diabetic patients in China. More detailed etiological studies of mucormycosis need to be done. We found the incidence of mucormycosis was tended to rise, which may be owing to the improvement of fungal diagnostic technology, health awareness and the visiting rate. In addition, diabetes incidence is on the rise worldwide, especially in China and India (Khan et al., 2020), which may be the reason for the increase in the incidence of mucormycosis in China.

For PM, the most common clinical symptoms were cough, fever, and hemoptysis/bloody phlegm. This result was similar to some previous studies (Skiada et al., 2011; Peng et al., 2019). Molecular-based assays can help to identify different fungal species and it can be used as a supplement to conventional diagnostic methods (Machouart et al., 2006; Springer et al., 2016). The study also found that laboratory results were not associated with patient outcomes. Most previous studies have not found a correlation between laboratory results and clinical outcomes in patients with mucormycosis (Patel et al., 2020; Son et al., 2020), and only a few studies have found that neutropenia may be associated with increased mortality (Spellberg et al., 2012). Neutropenia was not found in our patients with PM, it might not be an important risk factor for mortality in these patients.

The most frequent imaging findings of PM were patchy shadows, cavitation and pulmonary nodules in this study. According to the imaging findings, PM might be misdiagnosed as tumor or pulmonary aspergillosis. Nodule was more common in good outcome group. Lymph node enlargement were more common in patients with worse outcome. Many previous studies have found multiple nodules, reversed halo sign, and cavities associated with PM (McAdams et al., 1997; Marom and Kontoyiannis, 2011). Pulmonary nodules, halo sign and cavitation was also found in patients pulmonary invasive aspergillosis (Althoff Souza et al., 2006; Muldoon et al., 2016). The reversed halo sign has been considered an important clue to the diagnosis of PM, however, it has also been described in other pulmonary diseases, including invasive pulmonary aspergillosis, tuberculosis, organising pneumonia and malignancy (Sullivan and Rana, 2019). Therefore, early biopsy to establish the underlying cause is very important in patients with suspected pulmonary fungal infections.

Diagnosis of mucormycosis is challenging. In clinical practice, laboratory diagnosis of mucormycosis includes histopathology, direct examination and cultures (Skiada et al., 2020). Most patients in this study were diagnosed by histopathology. Many previous studies have also reported that histopathology was the main diagnostic method of mucormycosis (Prakash and Chakrabarti, 2019). In this study, Mucorales culture positive was found only in 13 patients. It was previously reported that the positive rate of Mucorales culture could reach 79% (Prakash and Chakrabarti, 2019). Which may be related to the low vigilance of doctors to invasive pulmonary mycosis and the absence of fungal culture for puncture specimens. More needs to be done to raise awareness of pulmonary mycosis among doctors.

In this study, twelve patients did not receive treatment and left the hospital against medical advice after diagnosis because of their critical condition and financial constraints. PM patients were treated with amphotericin B or posaconazole had better clinical outcomes, while untreated patients had poor outcomes. For rhino-orbito-cerebral and disseminated mucormycosis patients, there was no statistically significant difference in clinical outcome between different treatment strategies, which maybe related to the small number of cases. In addition, patients with rhino-orbito-cerebral mucormycosis who underwent surgery and antifungal therapy had better clinical outcomes. At present, treatment options for mucormycosis remain very limited. LAmB combined with surgery are strongly recommend as first-line therapy. Isavuconazole and posaconazole are also options as second-line agents (Cornely et al., 2019). For patients with PM, only 3 patients in this study were received pulmonary lobectomy, so no benefit of antifungal combined surgical treatment over antifungal therapy alone was observed. Bilateral lobe involvement occurred in 84.8% of patients in this study, which was similar to previous studies (Peng et al., 2019). Multifocal involvement limited the surgical options, early and effective antifungal therapy may be more important for patients with PM. The recommended combination of antifungal agents and surgical treatment as a treatment option for mucormycosis may be based on the presence of more rhino-orbito-cerebral mucormycosis in other countries. More researches may be needed to optimize treatment options for different types of mucormycosis. New treatment drugs or methods, such as combination of lipid amphotericin B and caspofungin or posaconazole, VT-1161, deferasirox in combination with a polyene, hyperbaric oxygen and so on, still deserve to be expected to improve clinical outcome (Skiada et al., 2018).

This study has some limitations. First, it was a retrospective observational study. Only some patients have complete follow-up data in our hospital, especially imaging data, and the follow-up of some patients could only be completed by telephone. Second, the detection rate of tissue culture was low, the pathological diagnosis was determined mainly by morphology and special staining. It is important to raise the awareness of doctors about fungal disease and the importance of tissue culture. Third, because mucormycosis is a relatively rare fungal disease in China (Liao et al., 2013), the number of cases in this study was limited. More larger-scale, multicenter studies of mucormycosis in the real world should be done.

## Conclusions

The most common clinical type of mucormycosis in China was PM. The most common risk factor was diabetes mellitus. Diabetic patients with clinical manifestations of febrile and hemoptysis, CT findings of nodules, cavities and bilateral lung involvement should be vigilant against PM. Early diagnosis and effective anti-mucor treatment are very important to improve the prognosis of patients with mucormycosis.

## Data Availability Statement

The original contributions presented in the study are included in the article. Further inquiries can be directed to the corresponding author.

## Ethics Statement

The studies involving human participants were reviewed and approved by Ethics Committee of West China Hospital, Sichuan University. Written informed consent for participation was not required for this study in accordance with the national legislation and the institutional requirements.

## Author Contributions

XL and JQ conceived of and designed the study. JQ and XL collected, analysed or interpreted data. JQ wrote the draft. All authors read, revised and approved the final manuscript.

## Funding

This study was supported by Sichuan Province Science and Technology Support Program of China (grant number: 2021YFS0170), 1•3•5 project for disciplines of excellence-Clinical Research Incubation Project, West China Hospital, Sichuan University (grant number: 2021HXFH032).

## Conflict of Interest

The authors declare that the research was conducted in the absence of any commercial or financial relationships that could be construed as a potential conflict of interest.

## Publisher’s Note

All claims expressed in this article are solely those of the authors and do not necessarily represent those of their affiliated organizations, or those of the publisher, the editors and the reviewers. Any product that may be evaluated in this article, or claim that may be made by its manufacturer, is not guaranteed or endorsed by the publisher.
